# Predicting psychological distress in advanced ovarian cancer patients during the COVID-19 pandemic

**DOI:** 10.1007/s00520-024-08675-0

**Published:** 2024-07-02

**Authors:** Mike Rennoldson, Michael Baliousis, Adam Potter, Ehab Ashraf, Ketan Gajjar

**Affiliations:** 1 Nottingham University Hospitals NHS Trust, Nottingham, UK; 2 University of Lincoln, Lincoln, UK; 3 Nottingham Trent University, Nottingham, UK

**Keywords:** Ovarian cancer, Distress, Fear of progression, Coping, Perceptions, Covid

## Abstract

**Purpose:**

This longitudinal study investigated distress rates in patients with advanced ovarian cancer during the COVID-19 pandemic and examined whether time, illness representations, and coping strategies predicted distress levels.

**Methods:**

UK patients with stage 3 or 4 ovarian cancer were recruited between September 2020 and March 2021. Data were collected at baseline (T0), 2 months (T1), and 4 months (T2) post-enrolment. Validated questionnaires assessed distress (anxiety, depression, PTSD, fear of progression) and predictors (coping strategies and illness perceptions), analysed via multilevel modelling.

**Results:**

Seventy-two participants returned a questionnaire at T0, decreasing to 49 by T2. High distress was observed, with over 50% of participants experiencing anxiety and depression consistently. Nearly 60% reported clinical levels of fear of progression at some point. PTSD rates resembled the general population. Although distress levels remained stable over time, some individual variability was observed. Time had minimal effect on distress. Coping strategies and illness perceptions remained stable. Threatening illness perceptions consistently predicted distress, while specific coping strategies such as active coping, acceptance, self-blame, and humour predicted various aspects of distress. Together, these factors explained up to half of the distress variance.

**Conclusion:**

The findings have implications for routine screening for distress and the inclusion of psychological treatment pathways in advanced ovarian cancer care. Addressing illness representations is crucial, with attention to informational support. Future research should explore the long-term effects of heightened distress and the effectiveness of interventions targeting illness perceptions. This study informs current clinical practice and future pandemic preparedness in cancer care.

## Background

The Coronavirus-19 pandemic posed serious challenges to people living with cancer, particularly affecting those with ovarian cancer. Ovarian cancer, often diagnosed at advanced stages due to subtle and easily overlooked symptoms, presents significant treatment challenges and a poor prognosis [1]. For example, the 5-year survival rate for women diagnosed at stages III or IV can be as low as 44%, reflecting the aggressive nature of the disease and the late stage at which it is typically detected [1]. These patients not only endure the typical stressors of the pandemic such as reduced social interaction and fear of COVID infection [2] but also face the compounded fears associated with aggressive treatment regimes, uncertain outcomes, and shortened life expectancy lived under social restrictions and fear of infection. Diagnostic and treatment services were disrupted as staff and facilities were redeployed to respond to the pandemic, leading to increased risk of poorer disease outcomes due to later cancer diagnosis, and delayed or restricted treatment choice. Supportive care was also disrupted with access to symptom management and psychological care restricted resulting in higher levels of unmet need [3]. Qualitative studies of the experience of cancer patients in the pandemic have identified fear of infection, social isolation, delays in care, the difficulty in having visitors, and uncertainty as key stressors [4–6].

There is evidence that the incidence of psychological distress increased in response to stressors posed by the pandemic, with rates higher in cancer populations than the general population [7, 8]. Psychological distress in psycho-oncology represents a multifaceted emotional state characterised by symptoms that may interfere with optimal functioning with common indicators including anxiety, depression, and other forms of psychological unease [9], influenced by a range of demographic, clinical, and social factors [10, 11]. In the general population, a meta-review of 18 meta-analyses of the prevalence of mental health conditions internationally during the pandemic found prevalence estimates of 26.9% for depression, 27.8% for anxiety, and 20.0% for PTSD [7]. For the cancer population, a meta-analysis of 24 studies during the pandemic found higher prevalence estimates, of 37% for depression and 38% for anxiety [8]. Crucially, the high levels of heterogeneity identified in these reviews highlights the importance of improving our understanding of distress in more specific cancer populations.

The small number of studies specifically addressing psychological outcomes in ovarian cancer patients during the pandemic supports the need for focused research. Advanced ovarian cancer where survival is poor, treatment onerous, and fear of progression ever present because of high rates of disease recurrence and frequent monitoring [1] is characterised by significant distress, and the effects of the pandemic might be expected to have been particularly acute. The small number of studies of psychological outcomes for people with ovarian cancer during the pandemic support this hypothesis. One large European study of individuals with various gynaecological cancers—50% of whom were patients with ovarian cancer—reported prevalence rates of 59% for anxiety and 51% for depression [12]. Another study specifically of people with ovarian cancer from the USA with a current or past diagnosis found prevalence rates of 51% for anxiety and 27% for depression [13]. These compare with prevalence estimates outside the pandemic from a meta-analysis of 23% for depression and 26% for anxiety in ovarian cancer patients while on treatment [14]. There is evidence that treatment disruption has increased fear of progression and distress in cancer patients [12, 15], and one UK survey reported that gynaecological cancer patients reported the highest rate of disruption to their care [16].

Fewer studies have examined other forms of distress which can be addressed via psychological intervention in cancer patients during the pandemic such as PTSD or fear of recurrence or progression — the fear, worry, or concern of the cancer returning or progressing [15]. A longitudinal study of PTSD symptoms in cancer patients in France during the pandemic found prevalence rates of people with moderate to severe symptoms ranged between 13 and 23% [17], which is comparable to those of the general population above. A scoping review of fear of recurrence and progression during the pandemic concluded that rates were higher than before the pandemic [15]. This raises questions about these aspects of psychological distress in advanced ovarian cancer.

Other than service disruption, there is little evidence about what predicts higher rates of distress in cancer patients including ovarian cancer. Understandably research designs during the pandemic have focussed on rapid data collection and dissemination, using cross-sectional methods, making inferences around predictors of distress particularly challenging. There is evidence that some demographic and disease characteristics were associated with worse distress including men, living alone, and a life expectancy of less than a year [18]. However, apart from the role demographic or clinical characteristics which are less amenable to intervention, the role of psychological predictors, which could be modified through psychological intervention, remains poorly understood. Further, there is little evidence regarding ovarian cancer specifically from outside of the pandemic also where ovarian cancer patients have often been grouped together with other cancer populations such as Breast or gynaecological cancer patients [19, 20] or evidence specific to ovarian cancer patients is limited to cross-sectional associations between factors such as rumination and intolerance of uncertainty with aspects of distress [21, 22].

Whilst there are few precedents to guide research into the psychological impact of the pandemic upon cancer patients, existing psychological theory can guide predictions. The self-regulatory model [23], which focuses on cognitive representations and coping, has strong empirical support in physical health. We might expect that cognitive representations, particularly of the controllability, consequences, and duration, of cancer might be affected by the pandemic and mediate the effect of the pandemic on cancer patients. We might also expect coping methods to have some effect on the level of distress. One study of coping found the most commonly used strategies in ovarian cancer during the pandemic were drawing on emotional support and distraction [24]. If illness perceptions and specific coping strategies were identified as reliable predictors of distress, this would open up new avenues for timely and targeted psychological intervention. Further, studies indicate that how patients perceive their illness and the strategies they employ to cope with its consequences can influence specific aspects of psychological distress differentially across cancer populations [10, 25]. Understanding the differential impact of illness perceptions and coping mechanisms on these dimensions of distress is crucial for developing targeted interventions.

Finally, from the study of stressful life events, long duration of a stressor has been found to predict distress in other contexts [26], but the evidence from the pandemic on the effects of time is mixed. Rates of PTSD symptoms have fluctuated with the lifting and return of lockdowns in France, though this relationship with lockdowns appeared to reduce over time [17]. There was little effect of time on distress found in a longitudinal study completed during and after the first wave of infections in Belgium [18]. Considering the specific features of ovarian cancer, establishing the trajectory of aspects of distress often targeted by psychological interventions, and their psychological predictors over time is important in developing the most efficient interventions. This study aimed to investigate rates of anxiety, depression, PTSD, and fear of cancer recurrence in people being treated for advanced ovarian cancer, how these varied over time, and the extent to which illness representations and coping were predictors of distress.

## Method

### Setting and participants

This research took place in the UK between September 2020 and May 2021. During this time, the UK experienced some of the most stringent containment and closure restrictions, and highest rates of coronavirus morbidity and mortality [27]. Our target sample was 80 based on prior studies in the area able to detect effects of magnitude that had clinical relevance [e.g., 25]. Participants were adult patients receiving treatment for stage 3 or stage 4 ovarian cancer in the UK, with life expectancy at least 3 months, and able to provide informed consent and complete the online or paper-based questionnaires in English. Patients were not eligible to participate if they had recurrent ovarian cancer, acute illness, receiving palliative treatment, or current diagnosis of severe mental illness. Participants were recruited in person either from one of eight cancer hospitals in England and Wales by their usual medical and nursing team, or via a UK wide ovarian cancer support charity, and were enrolled between September 2020 and March 2021. Throughout data collection, all nations of the UK were under some form of pandemic related social restrictions, with a full lockdown in place between December 2020 and March 2021.

### Design and procedure

This was a longitudinal study of distress patterns in ovarian cancer patients during the COVID-19 pandemic. Data collection occurred via self-report. At enrolment, following written informed consent procedures, participants opted to participate via online questionnaire hosted on the Qualtrics platform or via postal questionnaire. Data was collected at three timepoints: at baseline (T0), 2 months after enrolment (T1), and 4 months after enrolment (T2). Sample characteristics (demographics and clinical information) were systematically collected as detailed in Table 1 via self-report at baseline. At T0 and T2, participants completed a full set of validated questionnaire measures of anxiety, depression, fear of progression, PTSD, coping, and illness perceptions. To reduce participant burden and potentially improve participant retention at T1, participants completed a shorter questionnaire comprising only measures of anxiety, depression, and illness perceptions.

**Table 1:** Sample characteristics

Age (*n* = 69)	Mean age 69 (range 44–84)
Ethnicity (*n* = 70)	Asian/Asian British, *n* = 2
	Black/Caribbean, *n* = 1
	White British, *n* = 62
	White Irish, *n* = 4
	Other White, *n* = 1
Stage (*n* = 67)	Stage 3, *n* = 30
	Stage 4, *n* = 31
	Not sure, *n* = 6
Treatment (*n* = 67)	Chemotherapy only, *n* = 12
	Surgery then chemotherapy, *n* = 16
	Chemotherapy then surgery, *n* = 39
Time since diagnosis (*n* = 68)	Mean time since diagnosis 14 months (range 1–125)
Covid-19 prior to study intake, *n* = 63	Positive, *n* = 4
	Negative, *n* = 53
	Not tested, *n* = 6
Concurrent mental health treatment, *n* = 64	Currently receiving treatment, *n* = 11
	Not receiving treatment, *n* = 53

### Measures

Anxiety and depression were measured using the Hospital Anxiety and Depression Scale (HADS). This 14-item scale yields subscale scores for anxiety and depression, each ranging from 0 to 21. The psychometric properties of the HADS has been assessed extensively in cancer populations and been found to have good internal and test re-test reliability, validity, and sensitivity to change [28]. We used a cutoff of 8 and above to indicate caseness on each sub-scale. We chose a cutoff of 8 to enable comparison with other relevant literature.

PTSD was measured using the PTSD Checklist Civilian Version (PCL-C) which has shown high reliability and moderate validity in cancer populations [28]. The PCL-C has 17 items, scored from 1 to 5 which are summed. Various cutoff scores have been used in research — a cutoff of 44 has been found to maximise diagnostic efficiency [29].

Fear of progression was measured using the Fear of Progression questionnaire (FoP-Q-12). This 12-item scale are scored from 1 to 5 and produces a single score from 12 to 60. It has good internal reliability and concurrent validity in a cancer population with a score of 34 or above indicating “dysfunctional level of fear of progression” in cancer patients [30].

Illness perceptions were measured using the Brief Illness Perceptions Questionnaire (Brief IPQ) [31]. This has eight items concerning different aspects of illness representations including for example perceived control and consequences. Each item is scored from 0 to 10, of which three are reverse scored. Individual items can be used, or the items can be summed composite scale of overall illness threat used, which was the case in this study, resulting in a measure ranging from 0 to 80. The Brief IPQ has shown good reliability and concurrent, predictive, and discriminant validity in a range of health conditions, including cancer [31].

The Brief Coping Orientation to Problems Experienced scale (Brief COPE) [32] is a 28 item scale measuring 14 different coping methods. Each method is measured with the sum of two items which are rated on a scale from one (“I haven’t been doing this at all”) to four (“I’ve been doing this a lot”). It has acceptable reliability [32]. A score for each coping method is generated by summing its two items. A 14-factor structure has been confirmed in cancer populations [33].

### Analysis

Distress scores were summarised using means, and frequency counts of scores falling above clinical cutoffs. In preliminary analyses, we examined descriptive statistics and screened the data for input errors, outliers, assumption violations, and missing data [34]. We used multilevel modelling [35, 36] with maximum likelihood estimation. This approach is particularly suited to data where measurements are nested within individuals, as it allows for the analysis of changes within individuals over time while accounting for the lack of independence between repeated measures, and it is well-placed to cope with missing data in longitudinal studies.

In our models, fixed effects estimated the common intercept and the average change over time for all individuals, encompassing both level 1 (time-specific measurements such as anxiety and depression scores at each timepoint) and level 2 variables (patient-specific variables such as age and cancer stage). Random effects, on the other hand, allow these intercepts and slopes to vary among patients, thus acknowledging that different patients might start at different baselines and change at different rates over the course of the study. This inclusion of random effects at level 1 captures time-point-specific variability, whereas at level 2, it captures variability across patients.

To assess change in distress outcomes variables of anxiety, depression, PTSD, and fear of progression over time, models were fitted with random intercepts to test for variability in baseline distress levels across patients. Timepoint was introduced as a continuous level 1 predictor, assessing its impact on distress over the study period. To determine whether illness perceptions and coping styles predicted outcomes, we entered these as level 1 predictors and used established practice for model development [37, 38] to determine the final models using backward elimination (and not univariate analyses) where *P* > 1.57 while forcing in age and cancer stage, and timepoint with random intercepts only (baseline model). We also conducted sensitivity analysis without substance misuse as participants reported limited substance misuse.

We compared models using change in Δ*χ*^2^ (− 2LogLikelihood difference) and *R*^2^ compared to baseline [36] and computed *R*^2^ based on Rights and Sterba’s [39] recommendations. We explored the addition of random slopes but found insufficient observations to estimate these reliably for all outcomes. We computed 95% confidence intervals using percentile bootstrap estimation with 5000 samples. Level of significance *α* was 0.05. We used R software Version, 4.3.1 [40], with lme4 [41], lmertest [42], and r2mlm [43] packages.

## Results

### Sample description

The study sample consisted of 72 patients, predominantly female. Common treatments included chemotherapy alone, and combinations of surgery and chemotherapy, indicating a group undergoing significant medical intervention. An overview of the sample can be seen in Table 1.

### Attrition

Seventy-two participants returned a questionnaire at T0, 56 at T1, and 49 at T2 representing attrition rates of 22% at T1 (8-week follow-up), and 32% at T2 (16-week follow-up). High levels of missing data meant one participant was excluded at T1 and T2. We examined whether distress at T0 predicted drop-out and found no significant differences (|*t*s|≤ 1.26, *P*s ≤ 0.21, for anxiety, depression, and fear of progression; *W* = 434, *P* = 1, for PTSD [deviations from normality]). There was no difference in drop out between online and postal questionnaire completion (*χ*^2^ ≈ 0, *df* = 1, *P* = 1). There was also no association between missingness of predictor values and distress levels or participant characteristics (*Ps* > 0.06 for age; *Ps* > 0.1 for other variables). Therefore, we assumed data missing at random.

### Rates of distress

The results can be seen in Table 2. There were high rates of caseness in all four forms of distress at all timepoints, particularly anxiety which reached caseness in almost 50% of participants at all timepoints, and 65% of participants were above the anxiety cut-off at least once during the study.

**Table 2:** Mean scores and proportion reaching caseness for anxiety, depression, fear of progression, and PTSD at different timepoints

Distress measure	T0		T1		T2		Study period prevalence
	Mean (SD)	Point prevalence	Mean (SD)	Point prevalence	Mean (SD)	Point prevalence	
Anxiety	7.4 (4.2)	48.6% (*n* = 70)	7.4 (4.2)	50.0% (*n* = 54)	7.2 (3.9)	46.3% (*n* = 47)	65% (*n* = 47)
Depression	6.7 (4.0)	36.6% (*n* = 71)	6.1 (4.5)	29.6% (*n* = 54)	5.7 (3.8)	27.8% (*n* = 47)	50% (*n* = 36)
PTSD	31.0 (10.3)	15.9% (*n* = 69)	na	N/A	31.7 (11.8)	15.6% (*n* = 45)	21.7% (*n* = 15)
Fear of progression	33.2 (9.3)	40.3% (*n* = 68)	32.5 (8.1)	46.4% (*n* = 54)	31.5 (8.5)	42.9% (*n* = 47)	59.7% (*n* = 43)

Caseness cutoffs: anxiety HADS-A ≥ 8; depression HADS-D ≥ 8; PTSD PCL-C ≥ 44; fear of progression FoP-Q-12 ≥ 34

### Effect of time on distress

There was large variation in distress scores between patients (ICCs ≥ 63.8%) justifying use of multilevel modelling. We found little evidence of a systematic effect of time on any measure of distress. The mean scores for anxiety, PTSD, and fear of progression were stable over time (Δ*χ*^2^ ≤ 1.52, Δ*df* = 1, *Ps* > 0.05 for random intercept models; Δ*χ*^2^ ≤ 2.61, Δ*df* = 3, *Ps* > 0.05 for random intercept and slope models). The greatest change was a one point reduction in the mean depression score between T0 and T2, equating to approximately a half point reduction in score on the HADS-D every two months (Δ*χ*^2^ = 4.10, Δ*df* = 1, *P* = 0.04; *ß* =  − 0.47; CI − 0.92, − 0.04). This reflected very small change over time (Δ*R*^2^ = 0.009). We did not detect differences between patients’ depression trajectories overall (random slopes did not lead to further model improvement, Δ*χ*^2^ = 0.18, Δ*df* = 2, *P* = 0.91).

However, this stability of distress scores in the study sample as a whole masks some individual variability. While most participants had a stable trajectory over time on measures of distress, between 10 and 35% of participants moved above or below the caseness cut-off for each type of distress, during the study.

### Use of coping mechanisms and illness perceptions over time

Self-reported coping mechanisms were stable over the course of the study, measured 4 months apart at T0 and T2 (Δ*χ*^2^ ≤ 3.49, Δ*df* = 1, *Ps* > 0.05). The most and least commonly used coping approaches were the same at both time points. The most commonly used coping methods were acceptance, using emotional support from others, active coping, and self-distraction (means range 6.4–5.0). The least frequently used were avoidant coping mechanisms such as denial, self-blame, disengagement, and substance misuse (means range from 2.1 to 3.1). Participants reported substance misuse on seven occasions only, making this variable largely a constant. The largest change in use of coping strategy observed in the study was a 0.4 reduction in venting and use of emotional support between T0 and T2. Illness perceptions (IPQ) also showed little change overall between T0 (mean: 48.0) and T2 (mean: 46.2; Δ*χ*^2^ = 0.42, Δ*df* = 1,* P* = 0.52).

### Predictors of distress

Full models showed better fit compared to the baseline (with age, cancer stage, and timepoint; Δ*χ*^2^ ≥ 45.60, Δ*df* = 15, *Ps* < 0.05; Δ*R*^2^ ≥ 0.29). None of the full models offered any improvement over the final models following backward elimination (Δ*χ*^2^ ≤ 4.41, Δ*df* = 9–11, *Ps* > 0.05; Δ*R*^2^ ≈ − 0.01). The final models of the illness perceptions and coping, after controlling for age, cancer stage, and time, are shown in Table 3. Illness perceptions predicted all aspects of distress measured in our study, with bootstrap confidence intervals entirely above zero. Of the coping methods, more active coping predicted lower anxiety and depression, more substance misuse predicted more PTSD symptoms, more use of humour predicted, more anxiety-based distress (i.e., all of the aspects we measured except depression), more acceptance predicted less anxiety, and more self-blame predicted more anxiety and PTSD, with bootstrap confidence intervals entirely above zero also. These psychological factors alone explained approximately half of the variance (Δ*R*^2^) in ovarian patients’ anxiety, depression, and PTSD, and approximately a third of variance in patients’ fear of progression. The sensitivity analysis without substance misuse resulted in largely comparable findings except for PTSD where humour no longer predicted PTSD (*ß* = 0.86; CI − 0.02, 1.75).

**Table 3:** Final models for each distress measure, showing comparison with the baseline model containing age, cancer stage, and timepoint

	Anxiety	Depression	PTSD	Fear of progression
Compared to baseline				
Δ*χ*^*2*^(Δ*df*); Δ*R*^2^	65.42(6)***; 0.55	68.18(4)***; 0.58	59.80(5)***; 0.49	39.45(4)***; 0.28
Variables [*ß(CI)*]				
Age	0.02 (− 0.06, 0.09)	− 0.04 (− 0.11, 0.03)	− 0.02 (− 0.25, 0.21)	** − 0.43 (− 0.64, − 0.23)**
Cancer stage	− 0.64 (− 1.84, 0.57)	− 0.78 (− 1.93, 0.33)	0.33 (− 3.46, 4.12)	− 2.46 (− 5.89, 0.94)
Timepoint	− 0.24 (− 0.73, 0.23)	** − 0.56 (− 0.96, − 0.12)**	0.18 (− 1.01, 1.37)	− 0.21 (− 1.08, 0.68)
IPQ	**0.13 (0.08, 0.18)**	**0.15 (0.11, 0.20)**	**0.49 (0.34, 0.63)**	**0.36 (0.24, 0.47)**
Coping				
Self-distraction			− 0.72 (− 1.59, 0.17)	
Active coping	** − 0.50 (− 0.86, − 0.15)**	** − 0.45 (− 0.75, − 0.13)**		
Substance misuse	0.75 (− 0.15, 1.65)		**4.22 (1.57, 6.95)**	− 1.73 (− 3.71, 0.32)
Venting			− 0.80 (− 1.88, 0.26)	
Planning				0.58 (− 0.16, 1.32)
Humour	**0.36 (0.05, 0.66)**		**0.84 (− 0.01, 1.68)**	**0.82 (0.09, 1.50)**
Acceptance	** − 0.48 (− 0.87, − 0.09)**	− 0.34 (− 0.679, 0.002)		
Self-blame	**0.59 (0.18, 1.01)**	0.30 (− 0.07, 0.67)	**2.51 (1.23, 3.76)**	

*IPQ* Illness Perceptions Questionnaire score; *CI* confidence intervals (95%), provided instead of *P* values for coefficient estimates; Δ*χ*^2^(Δ*df*) =  − 2LogLikelihood difference between models (difference in degrees of freedom); Δ*R*^2^ = variance explained by full model in addition to baseline. Coefficient estimates in bold indicate CIs entirely above zero

***P < 0.001

## Discussion

This is the first longitudinal study of psychological distress in people being treated for advanced ovarian cancer during the pandemic. The study assessed four different forms of distress that might have been expected to affect patients with ovarian cancer and to be affected by the pandemic, and tested effects of psychological predictors of distress, based on a clear theoretical model. Our findings lend support to the hypothesis that rates of distress have been particularly elevated in people being treated for advanced ovarian cancer during the pandemic. We found a high period prevalence of anxiety and depression of 50% and above — higher than rates found in the general population and mixed cancer populations during the pandemic [8], and similar to the rates in the two studies focussed on gynaecological cancer populations [12, 24]. Rates of anxiety in particular were much higher than those found in ovarian cancer populations prior to the pandemic [14]. Our finding of 59.7% of participants experiencing dysfunctional levels of fear of progression at some point during the study is also higher than most estimates of rates fear of recurrence or progression in ovarian [1] and mixed cancer populations [44]. The key exception to this pattern is our finding of a period prevalence of 21.7% for PTSD was similar to levels found during the pandemic in the general population [7] and a mixed cancer population [17], and substantially lower than rates found in studies of PTSD in ovarian cancer or advanced gynaecological cancer [45, 46] which found rates above 30%.

We found no clear effect of time on rates of distress, other than a small reduction in depression scores. Neither effects of the chronicity of stressors nor effects of habituation to the stressors had detectable effects upon levels of distress overall. The lack of notable changes could indicate persistent high levels of distress, especially concerning anxiety and depression, which are likely exacerbated by the severe implications of advanced ovarian cancer, ongoing treatment challenges, and disruptions due to the pandemic. The stable distress levels mirror the consistency in threatening illness perceptions and coping strategies throughout the study period. This consistency may suggest that distress and at least its psychological drivers are not being adequately addressed in routine care. Another possible explanation is that time had two opposing effects: longer duration of pandemic stressors might increase distress, counter-acted by time allowing some participants to psychologically adjust. Furthermore, the absence of significant changes might also reflect a ceiling effect, where the measures of distress may not capture further increases in a population already experiencing high levels of distress. It is also important to note that there were a number of confounding effects that make it difficult to interpret this finding, particularly that participants commenced participation over a 6-month period, and while the general pattern was of significant lockdown restrictions during the study, these did vary over the course of the study. Therefore, there may be shorter term effects that the study was not sensitive to, both from social aspects of the pandemic and immediate coping responses.

The most consistent predictor of distress was threatening illness perceptions, which predicted all four forms of distress. Illness perceptions typically include perceived consequences, timeline, control, cause for concern, coherence, and symptoms attributed to the illness. The relationship suggested in our results underscores the powerful role that the appraisal of illness threats plays across psychological outcomes. Such perceptions, once established, appear to persistently shape emotional responses in ovarian cancer, perhaps more strongly and consistently than other cancer populations where early illness perceptions also set a trajectory for long-term psychological adjustment [10, 25]. Otherwise, only a subset of coping strategies seemed to predict the various aspects of distress. Together, these predictors accounted for approximately half of the variance in anxiety, depression, and PTSD, after controlling for age, stage, and timepoint but only up to a third of variance in fear of progression.

The specific coping strategies identified in the models were largely unsurprising. Active coping was associated with lower anxiety and depression scores, use of humour seemed to permeate higher levels in all three anxiety-related aspects of distress, self-blame predicted higher anxiety and PTSD scores, while more acceptance seemed to mitigate anxiety only. The role of humour seems largely unhelpful in the context of the current study and may mask distress at healthcare appointments or social contact. Together, with the relatively small number of coping strategies predicting distress, this may reflect an erosion effect of lockdowns on the effectiveness of coping strategies more broadly or ovarian cancer at stages 3 and 4.

Our findings illustrate the unique operation of the self-regulatory model in advanced ovarian cancer patients. While the effects of humour on distress seem prominent in our population, other results align with findings from diverse cancer populations outside the pandemic, for example, regarding the potentially pivotal role of illness perceptions across contexts [10, 25], the role of venting and self-blame in underpinning anxiety and PTSD in breast and head and neck cancer patients [19, 47], the protective effects of acceptance in breast cancer patients [48], or the limited role of coping in FOP relative to other aspects of distress in gynaecological cancers [49]. Specifically the weakening of approach coping such as planning and support-seeking in our study parallels cancer populations where invasive treatment erodes patients’ agency [25], and could be related to the COVID-19 context or the context of late stage ovarian cancer, thus warranting further investigation.

### Limitations

While the study had several strengths such as its longitudinal design and assessment of multiple forms of distress, there are important limitations. We followed each participant for four months, and this may have been insufficient to detect the effect of the duration of pandemic stress. Recruitment at the study centres was not consecutive, and we do not have data on which participants were approached and declined to participate. We cannot rule out systematic differences between our participants and the wider population of people being treated for ovarian cancer during the pandemic. Attrition between timepoints T0 and T1 was relatively high, although retention between T1 and T2 was good. Nevertheless, we could find no differences at T0 between participants who remained in the study, and those who dropped out on the variables we measured. It compares to attrition of 12% at 12 weeks and 26% at 24 weeks for a similar longitudinal study [18]. Further, the direction of causation is not clear in this longitudinal yet observational study.

### Future research

Researchers should turn next to investigating any scarring effects of elevated distress, both in newly diagnosed groups, and those who were receiving treatment during the pandemic. It will be important to assess whether individuals who experienced significant distress during the pandemic continue to be at greater risk for psychological challenges in the post-pandemic period. Understanding how these experiences may influence survivorship, quality of life, and disease progression in the long term is essential. Further investigation into psychological factors explaining fear of progression, beyond illness representations and coping, appears warranted on the basis of our findings. Finally, researchers should investigate the effectiveness of interventions that address illness perceptions in reducing distress in this population.

### Implications

Our study’s findings have implications for clinical practice now and pandemic preparedness in future. The high rates of distress suggest routine screening for distress, and psychological treatment pathways should be part of routine care for advanced ovarian cancer patients. The role of threatening illness perceptions suggests that careful attention to informational support, as well as a good standard of care, may be important in reducing distress in patients with advanced ovarian cancer. Future pandemic preparedness and clinicians supporting advanced ovarian cancer patients psychologically through treatment should consider the possibility that some coping mechanisms may lose their expected effectiveness and consider other ways of mitigating distress in this patient group.

## Conclusions

We tested theoretical mechanisms of distress and found that illness representations appeared important, but that time and coping strategies less so, in these conditions. These findings support the use of distress detection and psychological care pathways in advanced ovarian cancer, and further research into the stressogenic effects of the pandemic.

## Data Availability

Due to privacy and ethical concerns, the data supporting the findings of this study are restricted and not publicly available. Requests for access to the data can be directed to the corresponding author, subject to an evaluation of the request’s purpose and compliance with applicable privacy and ethical standards.
